# From Temporal Cell Arteritis to Giant Cell Aortitis Presenting as a Constitutional Syndrome: A Case Report

**DOI:** 10.7759/cureus.34181

**Published:** 2023-01-25

**Authors:** Marta Lopes, Marta Rocha, Marta Fonseca, Ana Monteiro

**Affiliations:** 1 Internal Medicine, Hospital Professor Doutor Fernando Fonseca, Amadora, PRT; 2 Internal Medicine, Hospital Professor Doutor Fernando Fonseca, Lisboa, PRT

**Keywords:** large-artery vasculitis, tocilizumab, constitutional syndrome, giant cell aortitis, giant cell arteritis

## Abstract

Giant cell arteritis (GCA) is the most common form of systemic vasculitis in adults, especially in patients over the age of 50. It manifests most commonly with an intense headache and visual symptoms. Although constitutional symptoms are also frequent in GCA, these can be dominant in 15% of patients at first presentation and 20% of patients when relapsing. Treatment with high-dose steroids should be initiated as soon as possible to rapidly control the inflammatory symptoms and prevent ischemic complications, the most feared being blindness from anterior ischemic optic neuropathy.

We present a case of a 72-year-old man who presented to the emergency department with a right temporal headache with retroocular radiation associated with scalp hyperesthesia, without any visual symptoms. The patient also reported low-grade fever, night sweats, anorexia, and weight loss over the last two months. The physical exam revealed a tortuous and indurated right superficial temporal artery, which was tender to palpation. The ophthalmological examination was normal. The erythrocyte sedimentation rate (ESR) and C-reactive protein (CRP) were elevated, and he also had inflammatory anemia with a hemoglobin of 11.7 g/L. Due to this clinical presentation as well as the elevation of inflammatory markers, the diagnosis of temporal arteritis was suspected, and the patient was started on prednisolone (1 mg/kg). A right temporal artery biopsy was performed on the first week after the initiation of corticotherapy and was negative. After treatment initiation, there was a remission of symptoms accompanied by a decrease and normalization of inflammatory markers. However, after steroid tapering, there was a reappearance of constitutional symptoms but without any other organ-specific symptoms, such as headache, visual loss, arthralgia, or other. The corticosteroid dose was increased to the initial dosage, but there was no improvement in the symptoms this time. After the exclusion of other causes of the constitutional syndrome, a positron-emission tomography (PET) scan was performed, which showed a grade 2 aortitis. The diagnosis of giant cell aortitis was assumed, and given the lack of clinical response to corticotherapy, tocilizumab was initiated with a resolution of constitutional symptoms as well as a normalization of inflammatory markers.

In conclusion, we report a case of temporal cell arteritis that further progressed to aortitis manifesting solely with constitutional symptoms. Furthermore, there was no optimal response to corticotherapy and no improvement with tocilizumab, therefore making this a case with a unique and infrequent clinical course. GCA is characterized by a wide variety of symptoms and organ involvement, and although it most frequently affects temporal arteries, it can be associated with aortic involvement that can cause life-threatening structural complications, highlighting the need for a high suspicion index for this condition.

## Introduction

Giant cell arteritis (GCA) consists of an immune-mediated inflammation involving the medium- and large-sized arteries. It is the most common form of systemic vasculitis in adults, being more frequent in patients over the age of 50. The pathogenesis of GCA is T-cell dependent and antigen-driven, and clinical subsets of GCA appear to result from variable cytokine expression [1,2].

The classic manifestations of GCA consist of headache, jaw claudication, polymyalgia rheumatica, and visual symptoms. Ischemic complications may result in permanent vision loss (up to 15%-25% of cases) if not recognized and treated early, making GCA an emergent diagnosis from an ophthalmological point of view [2,3]. As far as extracranial GCA is concerned, the aortic arch syndrome is uncommon as well as the involvement of femoral and brachial arteries, which can present as lower or upper extremity claudication [3,4].

Constitutional symptoms are very common in GCA and may include fever, fatigue, night sweats, anorexia, and weight loss. These features can be dominant in 15% of patients at first presentation and 20% of patients when relapsing [5].

Due to the wide-ranging phenotype, clinicians require a high index of suspicion for GCA because atypical presentations may delay diagnosis and lead to further complications [2]. As far as laboratory studies are concerned, workup includes erythrocyte sedimentation rate (ESR) and C-reactive protein (CRP) measurements, which are typically elevated with ESR above 50 mm/h. Although a sensitive marker, ESR is nonspecific. Furthermore, inflammatory anemia and mildly elevated liver enzymes may also occur [3].

The American College of Rheumatology (ACR) classification criteria were created in 1990 and consist of the following: (1) patient age > 50 years, (2) new-onset headache, (3) temporal artery abnormality (tenderness to palpation or decreased pulsation, unrelated to atherosclerosis of cervical arteries), (4) elevated ESR ≥ 50 mm/h, and (5) abnormal temporal artery biopsy. Although not diagnostic, the presence of three or more of the five criteria was associated with a sensitivity of 93.5% and a specificity of 91.2% for GCA when tested in a selected population of patients with vasculitis [4].

Temporal artery biopsy (TAB) is the gold standard for diagnosis, but it can be negative in one-third of cases. Since this condition can also involve large arteries, color Doppler ultrasound, magnetic resonance imaging (MRI), and positron-emission tomography (PET) scanning can be useful to assist in the diagnosis and evaluate the extent of the disease [2,3].

Treatment with high doses of glucocorticoids should be initiated as soon as possible in order to control the symptoms and prevent ischemic complications. However, over 80% of patients report significant side effects. Furthermore, the optimal dose and duration of steroid therapy are highly variable. In some cases, there is a need to add other immunosuppressant agents to control disease activity and reduce glucocorticoid toxicity. Of these agents, tocilizumab is the first to demonstrate an increased remission rate in both new and relapsing patients, being able to also lower the doses of glucocorticoids over one year in a large randomized controlled trial [6].

## Case presentation

We present a case of a 72-year-old male with essential hypertension and benign prostatic hyperplasia who presented to the emergency department on January 2018 with an intense right temporal headache with retroocular irradiation, which was pulsatile and associated with scalp hyperesthesia. The patient also reported fatigue, anorexia low-grade fever, night sweats, and non-quantified weight loss over the last two months. He denied any visual symptoms, such as ocular pain or loss of visual acuity. The physical exam revealed a tortuous and indurated right superficial temporal artery that was tender to palpation. An ophthalmology consultation was requested, and anterior ischemic optic neuropathy and other ophthalmological conditions were excluded. The cranial computed tomography (CT) performed in the emergency department was normal, and analytic evaluation showed an elevated ESR of 82 mm/h (normal range < 20 mm/h) as well as an elevated CRP of 20 mg/dL (normal range < 0.5 mg/dL). Furthermore, he also had an inflammatory anemia with a hemoglobin of 11.7 g/L (normal range: 13-17 g/L).

The diagnosis of temporal arteritis was suspected, and the patient was started on prednisolone (1 mg/kg). A biopsy of the right temporal artery was performed on the first week after the initiation of corticotherapy and was negative for GCA. Nevertheless, after the initiation of corticosteroids, there was a remission of symptoms accompanied by a normalization of inflammatory markers with an ESR of 18 mm/h and CRP of 0.4 mg/dL. After two months of 1 mg/kg prednisolone, slow steroid tapering was started. However, at the dose of 20 mg of prednisolone, there was a reappearance of constitutional symptoms, namely fatigue, anorexia weight loss, and night sweats with a new increase of ESR to 80 mm/h. Besides the constitutional symptoms, there were no other organ-specific symptoms, such as headache, visual loss, arthralgias, or others. The corticosteroid dose was increased to the initial dosage (1 mg/kg), but there was no improvement in the symptoms this time.

At this point, due to its persistence, other causes for the constitutional symptoms were excluded. The patient underwent a full-body CT scan, which was negative for neoplastic causes, as well as endoscopic exams, which were also normal. Given the high prevalence of tuberculosis in Portugal, the patient also underwent bronchoscopy and sputum examination, which were negative for this agent (negative direct examination with Ziehl-Neelsen stain and negative polymerase chain reaction for *Mycobacterium tuberculosis*). The patient was negative for the human immunodeficiency virus, and the venereal disease research laboratory (VDRL) test was also negative. The serologies for *Rickettsia*, *Borrelia*, and *Brucella* were also negative.

**Figure 1:**
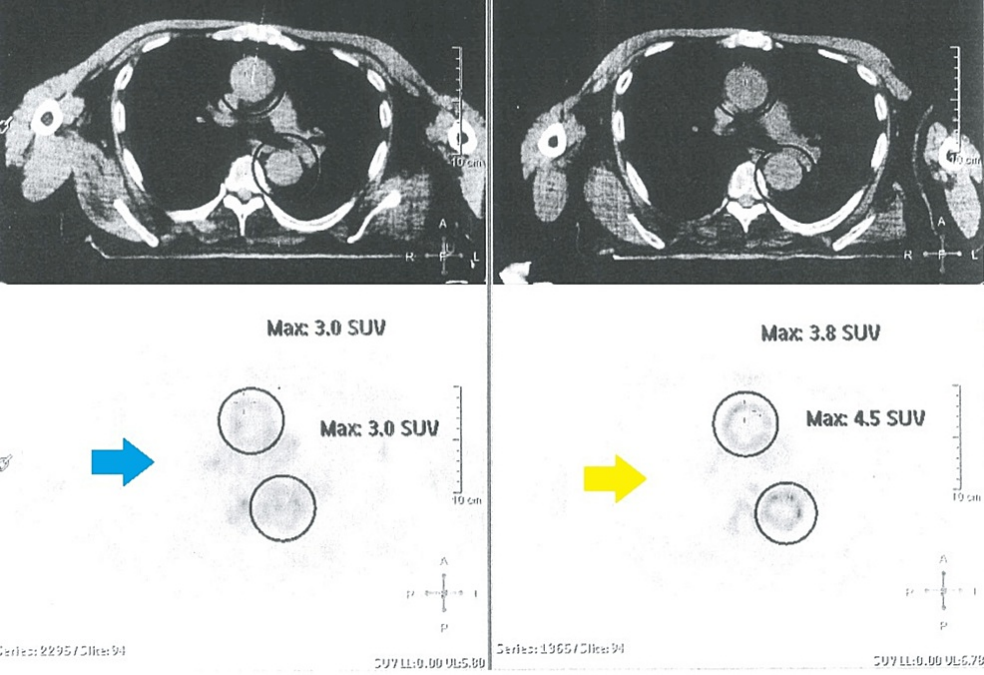
PET scans showing the evolution of aortitis before (yellow arrow) and after (blue arrow) treatment The first PET scan on the right shows grade 2 aortitis (yellow arrow), which was performed before treatment with tocilizumab. The second scan was performed after tocilizumab initiation with an improvement of aortic inflammation with grade 1 aortitis (blue arrow). PET: Positron-emission tomography.

**Figure 2:**
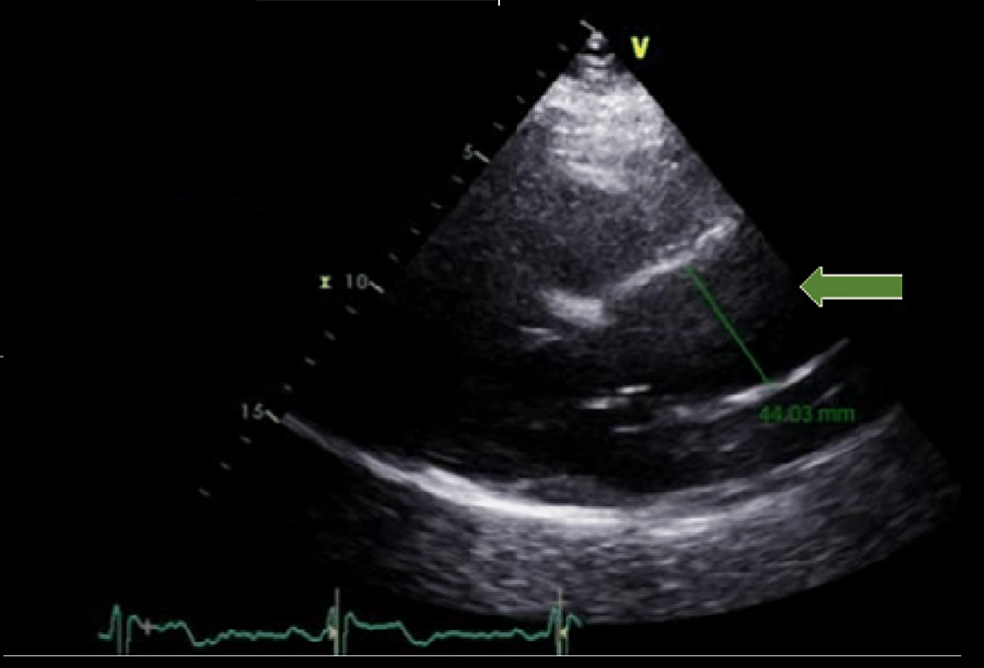
Parasternal long-axis view on transthoracic echocardiogram showing an ascending aortic dilation of 44 mm (green arrow)

Therefore, given the maintenance of constitutional symptoms that caused functional limitation to the patient, a PET scan was performed, which showed a grade 2 aortitis extending from the aortic arch to the emergency of the renal arteries (Figure 1). The transthoracic echocardiogram revealed an ascending aortic dilation of 44 mm (normal range: 17-33 mm) (Figure 2). Furthermore, the antinuclear antibodies were positive with a titer of 1/160, and the rheumatoid factor was negative. The anti-neutrophil cytoplasmic autoantibodies (p-ANCA) to bactericidal/permeability-increasing (BPI) protein were 1.6 UI/ml (positive cut-off value of 1.1 UI/ml). However, there were no manifestations specific to ANCA vasculitis, such as pulmonary-renal syndrome, skin vasculitis, chronic destructive airway disease, pulmonary nodules, or mononeuritis multiplex. The fact that the initial clinical presentation was very suggestive of GCA with temporal involvement which later progressed to aortic involvement, and also as the patient never had any signs or symptoms of ANCA vasculitis, led to the diagnosis of giant cell aortitis. Given the lack of clinical response to corticotherapy, tocilizumab was initiated (162 mg subcutaneous weekly), with a resolution of constitutional symptoms as well as normalization in inflammatory markers. A second PET scan was performed nine months after therapy with an improvement of inflammation in the previously affected areas with grade 1 aortitis (Figure 1).

## Discussion

GCA is the most common systemic vasculitis and may present with a myriad of signs and symptoms. In our patient, the initial symptoms were very suggestive of GCA without ophthalmological involvement as there were no visual symptoms and the ophthalmological exam was normal. The elevation of acute phase reactants such as ESR and CRP also corroborates this hypothesis. Even though it was performed one week after corticotherapy initiation, the TAB was negative. Although TAB is the gold standard for diagnosis, it can be negative in one-third of patients, so a positive TAB is not required for the diagnosis of GCA. Data has shown that the TAB needs to be done within two weeks of starting steroids or other treatments. Skip areas are not uncommon, and this could be the cause for the negative TAB in our case. Therefore, a large sample size is recommended when obtaining a TAB. Classic pathology finding is giant cell granuloma formation often near a disrupted internal elastic lamina [3].

Due to the highly suggestive symptoms and since the treatment should begin as soon as possible to prevent further complications, prednisolone was started at a high dose. The initiation of prednisolone was associated with symptom improvement and a decrease in inflammatory markers. The recurrence of only constitutional symptoms after tapering as well as their persistence even after a dosage increase made us think about other possible causes, such as neoplastic and indolent infections, which were therefore excluded.

The PET scan revealed a grade 2 aortitis, which led to the diagnosis of giant cell aortitis. Due to the clinical course, we can hypothesize that there was a possible progression from temporal arteritis to aortitis manifesting as constitutional symptoms. One of the complications is aortic aneurysm, and the patient already had aortic dilation on the echocardiogram. Patients with isolated extracranial GCA can present with nonspecific signs and symptoms, although vascular manifestations, mainly secondary to stenosis, may occur. Extracranial GCA is usually diagnosed by imaging such as PET scan, MRI, or CT angiography [7].

The positivity for p-ANCA BPI protein also makes this case unique, although it was only slightly above the reference range. The association between GCA and ANCA is not fully understood. While GCA is a granulomatous necrotizing vasculitis of large arteries affecting preferably cranial arteries as well as aorta and their branches, ANCA antibodies are involved in the pathogenesis of small- and medium-sized vessel vasculitis like granulomatosis with polyangiitis, eosinophilic granulomatosis with polyangiitis, and microscopic polyangiitis. The fact that the initial clinical presentation was very suggestive of GCA with temporal involvement, which later progressed to aortic involvement, is congruent with the medium-large vessel involvement in GCA. Also, the fact that the patient never had any signs or symptoms of ANCA vasculitis makes the diagnosis not probable. Although there have been some cases of ANCA positivity in GCA as well as overlap syndromes between these two entities, the pathological link is still not clear [8].

Finally, due to the lack of adequate clinical and laboratory responses to corticotherapy, tocilizumab was initiated. In patients treated only with steroids, there is a high rate of disease relapse (in 40%-80% of patients) [9]. Tocilizumab has been shown to significantly decrease the relapse rate and lower the steroid cumulative dose [6]. In our case, there was a positive response with symptom remission accompanied by a lowering of inflammatory markers and radiological improvement, which also supports the diagnosis of GCA aortitis.

## Conclusions

GCA is characterized by a wide variety of symptoms and organ involvement. Although it most frequently affects temporal arteries, aortitis can occur and usually presents with nonspecific symptoms, highlighting the need for a high suspicion index for this condition. Aortic involvement is likely to cause life-threatening structural complications, emphasizing the importance of a timely diagnosis. This case provides a report of temporal cell arteritis that further evolved to aortitis manifesting solely with constitutional symptoms. Furthermore, there was no optimal response to corticotherapy and no improvement with tocilizumab, therefore making this a case with a unique and infrequent clinical course.
